# MMpI: A WideRange of Available Compounds of Matrix Metalloproteinase Inhibitors

**DOI:** 10.1371/journal.pone.0159321

**Published:** 2016-08-10

**Authors:** Charuvaka Muvva, Sanjukta Patra, Subramanian Venkatesan

**Affiliations:** 1 Chemical Laboratory, Council of Scientific and Industrial Research-Central Leather Research Institute, Chennai, India; 2 Academy of Scientific and Innovative Research (AcSIR), New Delhi, India; 3 Department of Biotechnology, Indian Institute of Technology Guwahati, Guwahati, Assam, India; Florida State University, UNITED STATES

## Abstract

Matrix metalloproteinases (MMPs) are a family of zinc-dependent proteinases involved in the regulation of the extracellular signaling and structural matrix environment of cells and tissues. MMPs are considered as promising targets for the treatment of many diseases. Therefore, creation of database on the inhibitors of MMP would definitely accelerate the research activities in this area due to its implication in above-mentioned diseases and associated limitations in the first and second generation inhibitors. In this communication, we report the development of a new MMpI database which provides resourceful information for all researchers working in this field. It is a web-accessible, unique resource that contains detailed information on the inhibitors of MMP including small molecules, peptides and MMP Drug Leads. The database contains entries of ~3000 inhibitors including ~72 MMP Drug Leads and ~73 peptide based inhibitors. This database provides the detailed molecular and structural details which are necessary for the drug discovery and development. The MMpI database contains physical properties, 2D and 3D structures (mol2 and pdb format files) of inhibitors of MMP. Other data fields are hyperlinked to PubChem, ChEMBL, BindingDB, DrugBank, PDB, MEROPS and PubMed. The database has extensive searching facility with MMpI ID, IUPAC name, chemical structure and with the title of research article. The MMP inhibitors provided in MMpI database are optimized using Python-based Hierarchical Environment for Integrated Xtallography (Phenix) software. MMpI Database is unique and it is the only public database that contains and provides the complete information on the inhibitors of MMP. Database URL: http://clri.res.in/subramanian/databases/mmpi/index.php.

## Introduction

Matrix metalloproteinases (MMPs) are zinc-dependent endopeptidases which are implicated in various diseases. MMPs belong to the metzincin superfamily and are found in plants, vertebrates and invertebrates [1, 2]. MMPs are important homeostatic protease regulators of extracellular signaling and structural matrix environment of cells and tissues [3]. More than four decades ago, Gross and Lapierre [4] discovered MMP (type 1 collagenase). To date, 23 human MMPs have been reported (Table 1). On the basis of substrate specificity and homology, MMPs are classified into collagenases, gelatinases, stromelysins, membrane Type-MMP, matrilysins, enamelysin, metalloelastase and other MMPs [5, 6].

**Table 1:** Classification of matrix metalloproteinase enzymes.

Collagenases	Gelatinases	Stromelysins	Membrane Type-MMP	Matrilysins	Enamelysin	Others	Metalloelastase
MMP-1	MMP-2	MMP-3	MMP-14	MMP-7	MMP-20	MMP-19	MMP-12
MMP-8	MMP-9	MMP-10	MMP-15	MMP-26		MMP-21	
MMP-13		MMP-11	MMP-16			MMP-23	
		MMP-27	MMP-17			MMP-28	
			MMP-24				
			MMP-25				

MMPs are considered as promising targets for the treatment of many diseases such as arthritis, cancer, atherosclerosis, nephritis, aneurysms, tissue ulcers, and fibrosis [7]. Different research groups and pharmaceutical companies have made several attempts to develop inhibitors of MMPs. The first generation MMP inhibitors are limited by the poor bioavailability [8] (e.g., batimastat, D-5410, and Galardin), second generation inhibitors have side-effects [9] (marimastat) and the third generation inhibitors have no zinc-binding group and depth of the S1’pocket in most metalloproteases [10]. The effectiveness of the MMP class inhibitors require (i) functional groups like hydroxamate, carboxylate, thiolate, phosphinyl etc and (ii) capable of chelating the zinc(II) binding group.

Overall the domain architectures of various MMPs are significantly different. However, the active site geometries of the catalytic domain of different MMPs are similar. Current approaches for developing inhibitors consider secondary binding sites (exosites). These are referred to as regulatory sites, unique exosites have been proposed to be present in all MMPs [11–13]. Attempts have been made to develop peptide based inhibitors which bind secondary binding sites (exosites) of MMPs [14].

Numerous compounds have been synthesized by various research groups and also by pharmaceutical companies. These compounds have been screened to develop inhibitors of MMPs [15]. To search MMP Drug Leads, small molecule inhibitors and peptide based inhibitors, we developed an online database MMpI (Matrix metalloproteinases Inhibitors). It provides information on physico-chemical properties, biological activities (IC_50_ or K_i_ values) and hyperlinked to other databases. Overall, MMpI provides MMP Drug Leads, peptide, and small molecule inhibitors information.

## Materials and Methods

### Source of data

The primary data in the MMpI database are manually extracted from the full text of peer-reviewed scientific publications in various journals, such as *Journal of Medicinal Chemistry*, *Bioorganic and Medicinal Chemistry Letters*, *Organic Letters*, *Bioconjugate chemistry*, *European Journal of Medicinal Chemistry*, *Bioorganic and Medicinal Chemistry*, *Bioorganic Chemistry*, *The Journal of Biological Chemistry*, *Anti-Cancer Drugs*, *Journal of Enzyme Inhibition and Medicinal Chemistry*, *Nature Biotechnology*, *Biochimie*, *BioChemical Journal*, *Chemical and Pharmaceutical Bulletin*, *Journal of Agricultural and Food Chemistry*, *Matrix Biology*, *Biochemical Pharmacology*, *Bioconjugate Chemistry*, *Journal of Enzyme Inhibition and Medicinal Chemistry*. Although, the journals covered are not comprehensive, the selected volumes capture the high-quality information which is necessary for the development of database. From each publication the details of the biological activity of tested compounds, target protein and physico-chemical information are abstracted.

### Database architecture and web interface

MMpI is built on Apache HTTP server 2.4 with MySQL 5.6 at the back end, and the PHP 5.5 and JavaScript at the front end. Apache, MySQL, and PHP are preferred as these are open-source softwares and platform independent.

## Results

### Description of MMpI database

**Fig 1:**
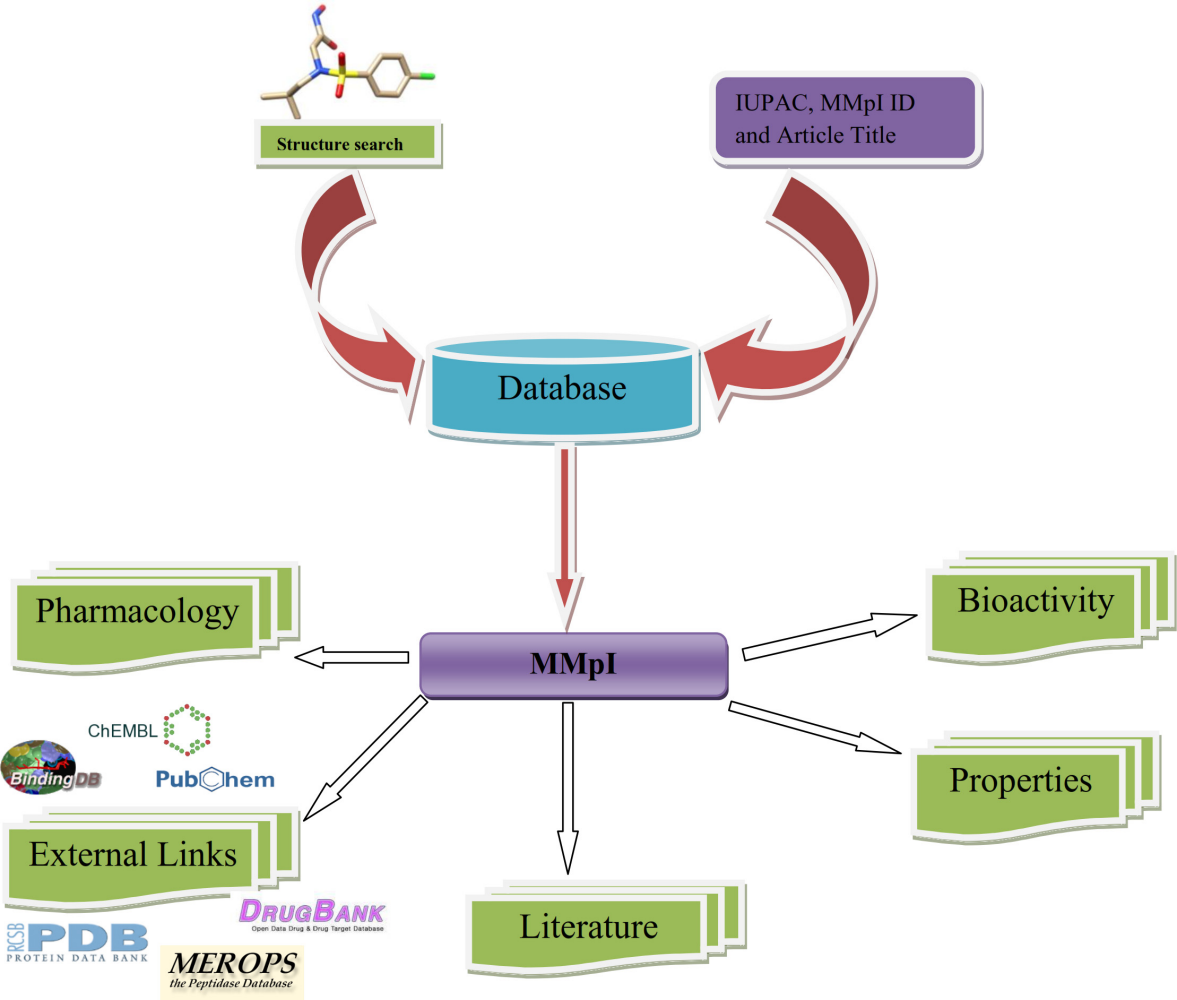
A schematic representation of the MMpI Pipeline.

**Fig 2:**
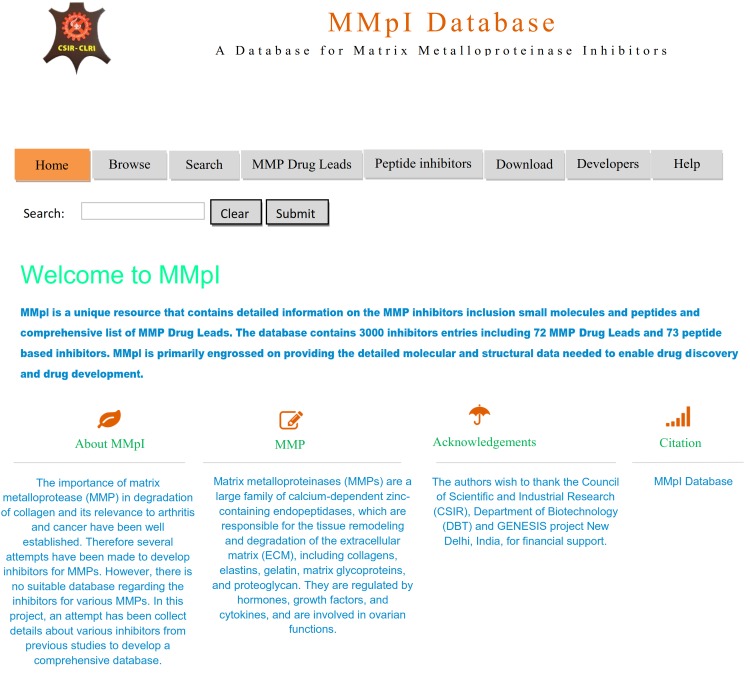
Home page of the MMpI database.

The MMpI database is openly accessible via a simple, user friendly interface at **http://clri.res.in/subramanian/databases/mmpi/index.php**. It is a web-based and platform-independent database with ~3000 inhibitors including ~72 MMP Drug Leads and ~73 peptide based inhibitors. The pipeline of MMpI database is presented in Fig 1 and the home page of the MMpI database is shown in Fig 2. For example, user can retrieve potential compounds by employing a keyword search of the database using IUPAC name, derivative type, disease based, MMpI identifiers, and type of MMP and title of research article of interest.

**Fig 3:**
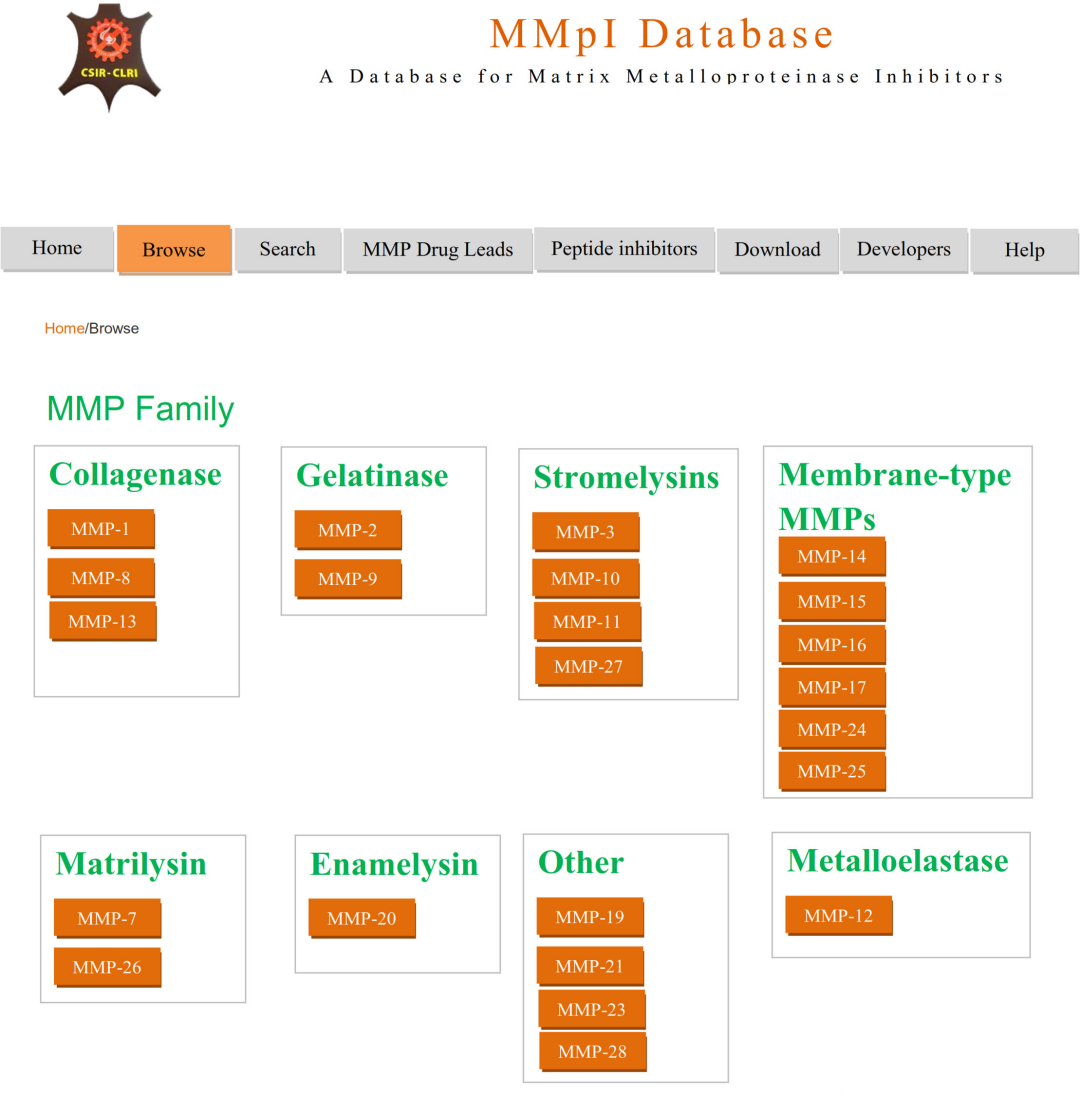
Interface of the MMpI database browser page.

**Fig 4:**
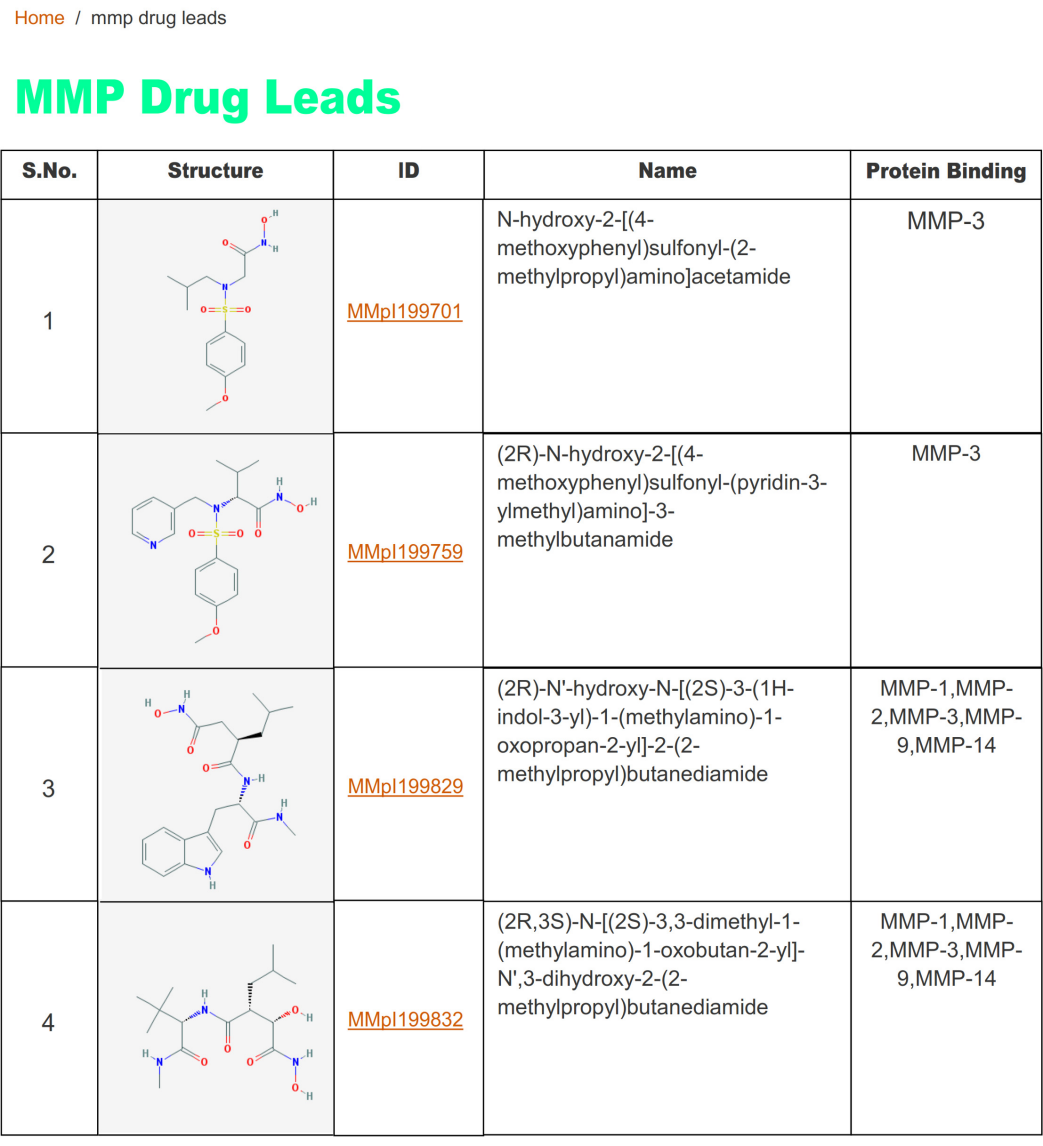
Screenshot of the MMpI database showing MMP Drug Leads.

The browser interface shows the classification of matrix metalloprotease. Using the interface, the investigator can derive the information (Fig 3). A table view of MMP Drug Lead molecules is also provided in MMP Drug Leads interface, with structure, MMpI ID, IUPAC name and relevant binding protein (Fig 4). Users can go to compound record card to access further information, such as structure, bioactivity (IC_50_ or K_i_ values), and physico-chemical information.

**Fig 5:**
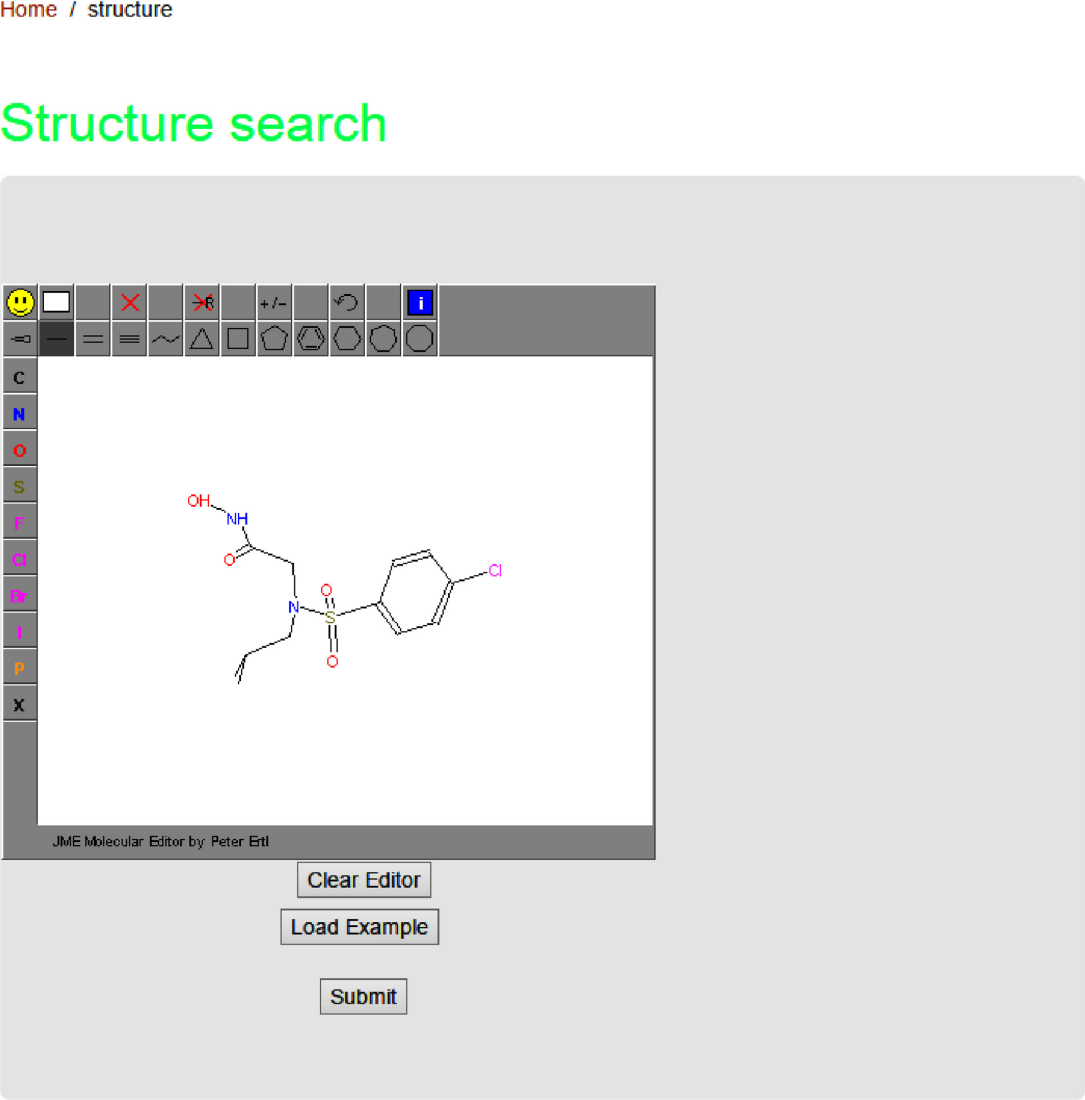
Choice of sketchers allows the user to draw a structure of interest and search the database for similar compounds.

This Database has a feature to search for a particular compound of interest and to retrieve information about the compound, or closely related compounds. The structure interface provides the JME molecular editor drawing tool [16]. It is possible to sketch a structure of interest. A compound similarity search of the database can be carried out to retrieve inhibitor which is similar to the input structure (Fig 5).

**Fig 6:**
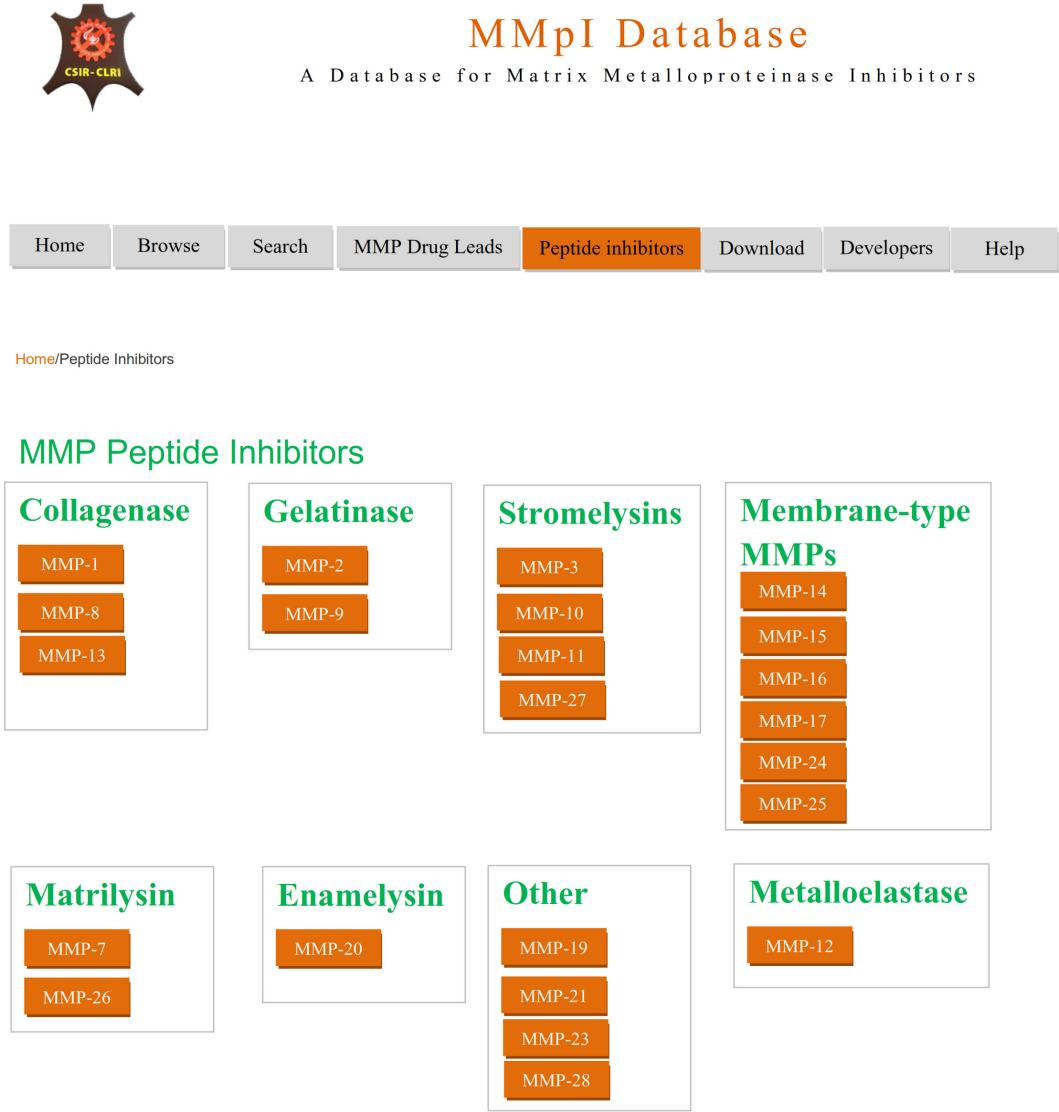
Screenshot of the MMpI database showing MMP peptide inhibitors.

The interface for peptide inhibitors and triple helical peptide inhibitors show the classification of matrix metalloprotease. Based on this, the researchers can view the database and retrieve information on the peptide inhibitors (Fig 6). This interface provides the MMpI database unique id for peptide inhibitor, type of MMP, peptide sequence, bioactivity (IC_50_ or K_i_ values) and journal information.

**Fig 7:**
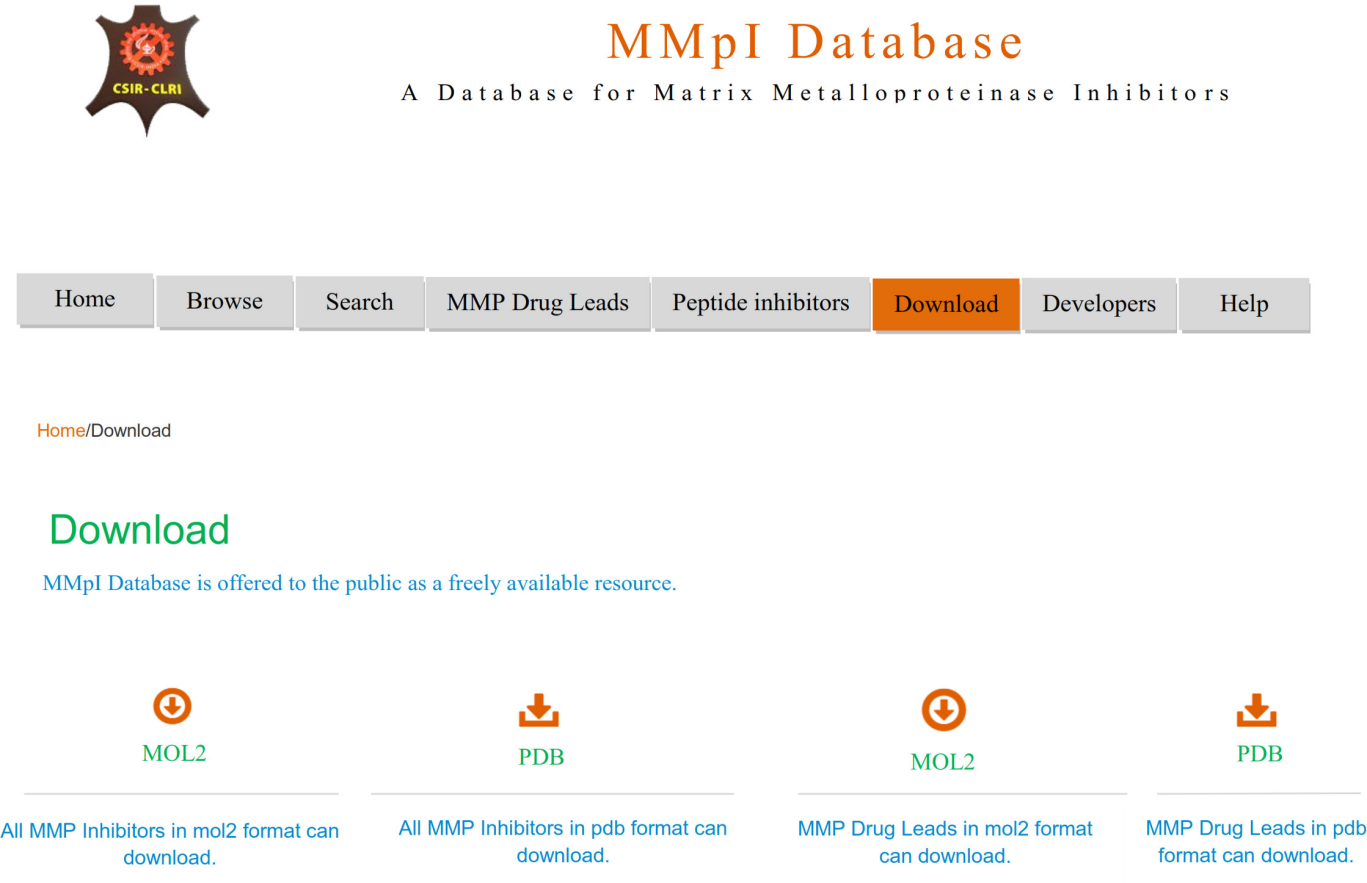
Interface of the MMpI database download page.

The download interface allows the investigator to download the MMP inhibitors and MMP Drug Leads in pdb and mol2 formats (Fig 7). The MMP inhibitor and drug lead files in pdb and mol2 formats were optimized using Python-based Hierarchical Environment for Integrated Xtallography (Phenix) software [17]. This optimized compound can be used for drug design, docking or screening studies.

### Example

**Fig 8:**
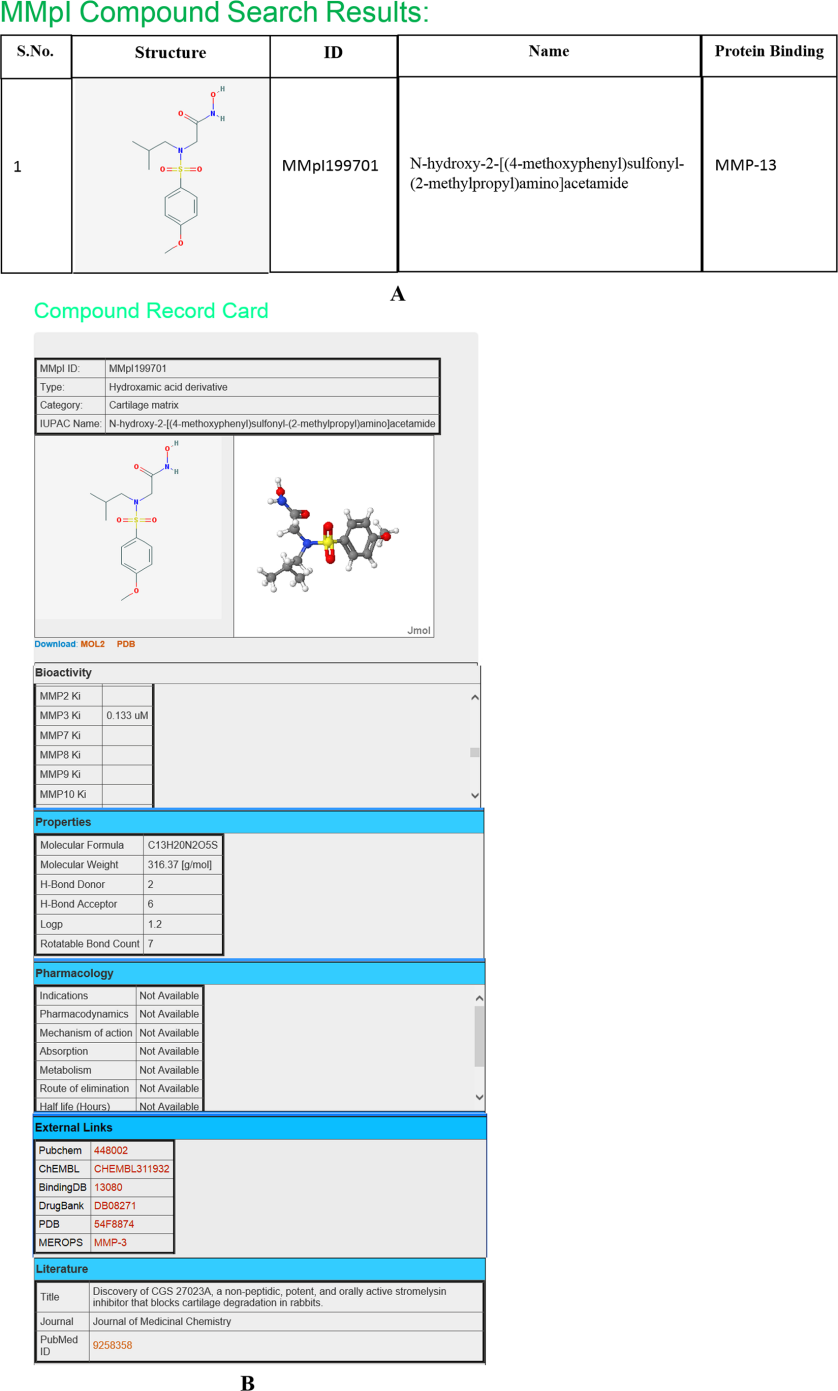
A screenshot montage of the MMpI database showing. (A) Compound search results. (B) MMP compound record card.

It is expected that researchers look for essential and specific small molecule inhibitors or peptide inhibitors or triple helical peptides for MMP. As an example, to get the structure or analogs of hydroxamic acid inhibitors of stromelysins, one can search via search box or can sketch using JME tool which is incorporated in this database (Fig 5). Upon submission, the MMpI compound search results in a page with suitable structures. Then the researcher can select the compound of interest and the page will redirect to MMpI, compound record card page. This page provide further details about MMpI ID, IUPAC name, type of inhibitor and structures (2D and 3D) visualization in 2D and 3D using Jmol [18]. In addition, it is also possible to download the 3D structure in pdb and mol2 formats. The compound record card also covers bioactivity (IC_50_ or K_i_ values), physico-chemical properties and pharmacological information. In addition, record card page provides cross-references to other resources like Pubchem [19], ChEMBL [20], Binding DB [21], DrugBank [22] PDB [23] and MEROPS [24]. Finally the report card contains a link to the source of the journal from where the information is retrieved (Fig 8A and 8B).

## Discussion

MMpI is a web-accessible database that offers quantitative chemical, physical, pharmaceutical and biological data about thousands of well-studied drug leads, inhibitors and peptide inhibitors of MMP. MMpI is primarily focused on providing detailed molecular data needed to facilitate drug discovery and development. MMpI is unique, not only in the type of data but also in the level of integration and depth of coverage. In addition to its extensive coverage of small molecules and drugs, it is the only public database that provides information on the inhibitors of matrix metalloproteinases. MMpI also supports an extensive array of visualizing, querying and search options. It is hoped that MMpI will serve as a useful resource to research community.

### Further development

We will try to incorporate the new releases as soon as they will be available in the public domain.
